# “Married with children” the influence of significant others in TTO exercises

**DOI:** 10.1186/s12955-015-0276-7

**Published:** 2015-07-02

**Authors:** F.E. van Nooten, N.J.A. van Exel, X. Koolman, W.B.F. Brouwer

**Affiliations:** Institute of Health Policy & Management (iBMG) and Institute of Medical Technology Assessment (iMTA), Erasmus University Rotterdam, Rotterdam, The Netherlands; Department of Health Sciences, VU Amsterdam, Amsterdam, The Netherlands; iBMG/iMTA, Erasmus University Rotterdam, PO Box 1738, 3000DR Rotterdam, The Netherlands

## Abstract

**Background:**

Which responder characteristics influence TTO scores remains underexplored. More research is needed in order to understand (differences in) TTO scores, but also in the context of generating representative health state valuations for some population. Previous studies have found age, gender, marital status and subjective life expectancy to influence the number of years traded off.

**Objective:**

This study aimed to investigate which other responder characteristics influence TTO responses, with an emphasis on consideration of significant others, such as partners and children.

**Methods and Design:**

We performed a web-based survey in a representative sample of the Dutch general public (aged 18–65). Data on demographics, health status and expectations about future length and quality of life were gathered. Respondents valued three distinct health states using TTO.

**Results:**

A total of 1067 respondents completed the questionnaire. Sixty percent of respondents had children and 49 % were married. The mean number of years traded off increased with severity of health states. Higher age and living together were positively associated with number of years traded off. Increases in subjective life expectancy, having children and being male (were negatively associated with the number of years traded-off.

**Conclusion:**

Age, gender and subjective life expectancy, living together and having children were significantly associated with TTO responses. Consideration of significant others in TTO exercises thus may be important in understanding (differences in) TTO responses and when drawing representative samples from the general public.

**Electronic supplementary material:**

The online version of this article (doi:10.1186/s12955-015-0276-7) contains supplementary material, which is available to authorized users.

## Introduction

A commonly used method for deriving health state valuations is the time trade-off (TTO) method. These health state valuations are important in the context of constructing QALY scores for different health profiles, which can be used in cost-effectiveness analyses of health technologies. The TTO method is widely used, e.g. to derive national tariffs for often used measures like the EuroQol EQ-5D instrument (e.g. [1, 2]).

In a TTO exercise, respondents are asked to indicate their indifference between two streams of health that differ in terms of length and quality of life. Commonly, one stream entails an imperfect health state that will last for a fixed period of time X (often 10 years). The second stream entails perfect health, but this is enjoyed for a shorter period of time than X. The TTO exercise then requires respondents to reveal their point of indifference between two streams, usually by varying the number of years in perfect health.

TTO exercises thus provide a relatively simple means of deriving health state valuations, allowing QALY scores for health states to be used in health economic evaluations. Nonetheless, the TTO method has been shown to be prone to several types of biases [3], its operationalization may influence results [4], and the way in which the answers of respondents usually are interpreted has been argued to be too simplistic (e.g. not accounting for time preferences for health) [5]. Moreover, it has been shown that certain respondent characteristics like age, gender and marital status can influence the number of years traded-off (i.e. health state valuations) [6]. This emphasizes that it is important to know which characteristics of respondents influence TTO scores. Knowledge and understanding of the characteristics of respondents that influence TTO scores is important for several reasons: (i) when comparing health state valuations between respondents, populations or studies, (ii) for sampling purposes, when the aim is to generate representative health state valuations for a specific population, and (iii) to increase understanding of underlying considerations and mechanisms driving observed health state valuations.

Three clusters of responder characteristics influencing TTO scores may be distinguished: (i) the demographic characteristics age, gender, and marital status [6]; (ii) health status [7, 8] and health related characteristics (such as BMI) [9]; and (iii) subjective reference points for future length and quality of life [10–12].

It is conceivable that other responder characteristics are associated with the number of years traded in TTO exercises as well. TTO decisions may be especially affected by consequences on significant others, such as partners, children and family members. For instance, living shorter by trading off more life time may be considered less attractive when one has young (dependent) children, like observed by Van Der Pol & Shiell [13]. They found that recent mothers valued health states differently than the general population. Similarly, Devlin and colleagues found that females in a household with children provided significantly higher health state values [7]. Health state valuations may also be influenced by consideration of the consequences on partners and family members of living shorter or in poor health. For instance, Matza and colleagues [14] observed that individuals with a caregiver role were less willing to trade off time to improve their health status than non-caregivers. Conversely, consideration of others could also lead to increased trading of years, in order not to be a burden to others as recipient of informal care [15].

While former studies have demonstrated the influence of marital status and having children on health state valuations using TTO, it remains unclear whether these are separate effects or that marital status perhaps combines the influence of having a partner and having children on TTO scores [6]. This paper reports the results of a study that aimed to investigate the association of a broad range of responder characteristics with TTO scores, with a special emphasis on the consideration of significant others and on disentangling the influence of having a partner and having children on TTO scores.

## Methods

The questions for this study were part of a larger web-based questionnaire which was administered online by a professional survey company to a representative sample of the Dutch general public in terms of gender and age (in the range 18–65 years). A minimum completion time of 15 min was set, based on a pilot test of the survey. Respondents who completed the survey in less than 15 min were identified as speeders and excluded from the analyses. A total of 1223 respondents participated in the online survey; 156 (12.8 %) respondents were disregarded because of speeding. For the remaining 1067 respondents, mean response time for the questionnaire was 27.8 min (SD 8.2; range 15–63).

At the time of the design of this web-based TTO exercise, neither a universal web-based TTO protocol existed nor a standardized EuroQol web-based TTO. Hence, one was created. The questionnaire first asked about general responder characteristics, such as age, gender, marital status, nationality, level of education, having children, number of children and age of the youngest child. Next, respondents were asked about their current health (using VAS and the EQ-5D) and whether they currently have or have had a chronic or serious condition. In addition, they were asked the following question: “If, due to some illness, you had to choose between a shorter life in good health and a longer life in poorer health, what would you choose at this moment?”

Then, respondents were asked to value six health states. Five of the six health states were described using the three level EQ-5D descriptive system. The five health states were own health (as previously indicated by the respondent), perfect health and three imperfect health states (EQ-5D profiles 21,211, 22,221 and 33,312 - see Additional file 1). The latter were chosen to represent a broad range across health states and identical to those used in previous studies [11, 16], also to facilitate comparisons. The sixth state was labeled as ‘dead’. To familiarize the respondents with the health states and tasks, they were first asked to rank these six health states and then to rate them using a visual analog scale ranging from 0 (worst imaginable health state) to 100 (best imaginable health state). Finally, respondents were asked to perform TTO exercises for the three imperfect health states mentioned above. The three TTO exercises (see Additional file 2) were presented to respondents in the order in which they had ranked them in the ranking exercise, with the highest ranked health state first.

In the TTO exercise respondents were first asked to choose between living 10 years in a specific imperfect health state followed by death (option A), or living 10 years in a perfect health state followed by death (option B). They could also indicate to be indifferent between the two (Option C). If the dominated option A was chosen, respondents needed to confirm this preference or choose again. If option B was chosen, they were asked to choose between living 10 years in the imperfect health state, after which they would die (option BA), living 5 years in a perfect health state, after which they would die (option BB), or being indifferent between these options (option BC). If respondents chose option BA they were shown a slider ranging from 0 to 10 years and asked to indicate how many years in perfect health would be equivalent to living 10 years in the imperfect health state presented to them. If respondents chose option BB they were shown a slider ranging from 0 to 5 years and asked to indicate how many years in perfect health would be equivalent to living 10 years in the imperfect health state. The slider in options BA and BB allowed indicating years with one decimal level of precision. Finally, respondents were asked to confirm that they were indifferent between living *X* years in perfect health and 10 years in the imperfect health state, with *X* taking the slider value in case of options BA and BB or 5 in case of option BC. If respondents immediately chose option C they were asked to confirm that they were indifferent between living 10 years in imperfect or perfect health state. In all cases, if respondents did not confirm their choice, they returned to the beginning of the question.

In this TTO exercise we did not include a protocol for states perceived as being worse than dead, since we felt this would be too complex in the context of self-completed online questionnaires [11]. The ranking of the six states indicated that especially the worst health state (33312) was ranked as worse than dead by some of the respondents (around 9 %). Since we did not have a separate valuation exercise for states considered to be worse than dead, we included all TTO responses obtained in the regular TTO exercise in the analyses. No responses were excluded therefore.

After the three TTO exercises, respondents were asked whether they had related the 10 year time frame to their own life while answering the questions, and whether they had assumed the 10 years period to start immediately. Moreover, we asked respondents whether they had considered specific moments in time that they wanted to reach (e.g. an anniversary or specific age) within that time frame. If so, they were asked to indicate which moments.

Finally, respondents answered some questions regarding their subjective life expectancy and their quality of life expectations at the ages of 60 (if aged 59 or less), 70, 80 and 90 years, using the EQ-5D as done before [10, 17, 18].

A number of variables were constructed for the analyses. The number of years sacrificed out of the remaining 10 was calculated (10 minus the minimum number of years required in perfect health). Subjective life expectancy (SLE) was calculated by deducting the actual age from expected age of death. The utilities for quality of life expected at 60, 70, 80 and 90 years were calculated using the expected EQ-5D health profiles and the Dutch EQ-5D tariffs [2]. Body mass index was calculated by dividing the weight responders provided by the square of their height (in meters).

Analyzing the data, we first ran a regression model including explanatory variables that used in previous research, as discussed in the introduction: age, gender, marital status, educational status, own health and subjective life expectancy. Next, the contribution of three additional types of responder characteristics was investigated, i.e.: demographics (gender, age, highest education, marital status, children yes/no, number of children, age youngest child), health (VAS; chronic illness; serious illness; weight) and expectations (subjective life expectancy and quality of life at the ages of 60, 70, 80 and 90). First, regressions were performed per TTO question per type of responder characteristic, giving in total three regressions. If a variable was statistically significant (*p* < 0.05) in at least one of the regressions it was included as explanatory variable in the final model. As the data was not normally distributed, bias corrected confidence intervals were obtained using a non-parametric bootstrap procedure using 10,000 replications.

## Results

### Responder characteristics

A total of 1223 respondents completed the questionnaire of which 156 (12.8 %) were removed because of speeding through the questionnaire. The remaining 1067 respondents were included in the analyses. Table 1 shows that the mean age of the sample was 43 years, half of the respondents were male and mean VAS score was 75. Sixty percent of the total sample had children, varying between 8 % among those who were single and 86 % among those who were married. Twenty eight percent of the respondents indicated to have (had) a serious condition and 36.6 % indicated to have (had) a chronic condition. The average BMI was 26.4 and 19.1 % indicated to be overweight.

**Table 1:** Demographics of the sample (*n* = 1067)

Age (mean, SD, range)		43.2 (13.64) 18–65
Gender (male) (%)		50.2 %
Education (%)	Lower	15.4 %
	Middle	53.7 %
	Higher	30.9 %
Marital status (%)	Married	49 %
	Living together	15.3 %
	Divorced	8.5 %
	Widow(er)	2.2 %
	Single	21.5 %
	Don’t want to reveal	3.5 %
Children (yes) (%)		60.2 %
Number of children (mean, SD, range)		2.1 (0.94) 1–11
Age of youngest child in years (mean, SD, range)		17.1 (11.4) 1–44
Dutch (%)		98.6 %
Employed (%)		47.3 %
Current quality of life EQ-5D VAS (mean, SD)		75.0 (16.59)
Current quality of life EQ-5D utility (mean, SD)		0.85 (0.23)
Do you have ever had a serious condition? (yes) (%)		28.2 %
Do you have a chronic disease? (yes) (%)		36.6 %
BMI (mean, SD)		26.4 (5.08)
Overweight? (yes) (%)		56.1 %
Obese? (yes) (%)		19.1 %
SLE (mean, SD)		37.8 (17.21)
Quality of life at years 60 (EQ-5D utility (mean, SD)) (*N* = 921)		0.77 (0.27)
Quality of life at years 70 (EQ-5D utility (mean, SD))		0.69 (0.30)
Quality of life at years 80 (EQ-5D utility (mean, SD))		0.51 (0.37)
Quality of life at years 90 (EQ-5D utility (mean, SD))		0.32 (0.42)

In total, 56 % of the respondents indicated to have a preference for a shorter life in perfect health over a longer life in imperfect health. Irrespective of their living situation (single or in a partnership), 63 % of the respondents without children and 49 % of the respondents with children would prefer quality of life over longevity. In addition, irrespective of having children, 50 % of the responders who were married chose longevity over quality of life while this was 36 % of the unmarried respondents who did have a partner.

In total, 16 % of respondents indicated to wish to reach a specific moment in time, of which 68 % had children.

### Numbers of years traded off in the health states

**Fig. 1:**
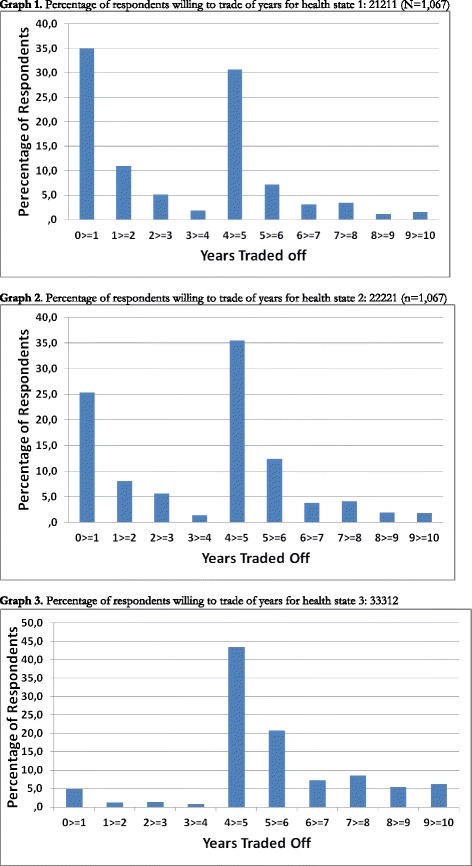
Years traded per health state among traders

The average number of years respondents wanted to trade off was 3.16 years for health state 1 (i.e. the highest ranked one), 3.80 years for health state 2 and 5.63 years for health state 3 (Table 2). Figure 1 shows the distribution of years traded off in each of the health states. Table 2 shows that respondents who indicated to have a preference for a shorter life in perfect health over a longer life in imperfect health, indeed traded off significantly more years than respondents who preferred to live longer in imperfect health (*p* < 0.05).

**Table 2:** Years traded off of respondents who prefer to live shorter in perfect health compared to respondents who prefer to live longer in imperfect health

	Mean (SD)	Shorter in perfect health (*n* = 599)	Longer in imperfect health (*n* = 468)	*P* ^a^
Health State 1 (21211)	3.16 (2.58)	3.65 (2.48)	2.53 (2.56)	0.00
Health State 2 (22221)	3.80 (2.56)	4.43 (2.31)	2.99 (2.65)	0.00
Health State 3 (33312)	5.63 (2.01)	6.00 (1.79)	5.15 (2.17)	0.00

^a^independent samples *T*-test

Table 3 shows the results of the model that included variables also used in previous research (model 1). A negative sign means that respondents were willing to give up less years in the TTO exercise. In this model age (negative), marital status (negative) and subjective life expectancy (SLE) (negative) were statistically significantly associated with the number of years traded off. Education level and health measured by VAS were not statistically significantly associated with the number of years traded off.

**Table 3:** Results (dependent variable: Years traded-off)

	Model 1			Model 2			Model 3		
	Average marginal effect	Bias corrected 95 % CI		Average marginal effect	Bias corrected 95 % CI		Average marginal effect	Bias corrected 95 % CI	
Age	**−0.044**	**−0.057**	**−0.032**	**−0.044**	**−0.053**	**−0.036**	**−0.040**	**−0.049**	**−0.032**
Male	**0.306**	**0.092**	**0.521**	**0.313**	**0.161**	**0.466**	**0.275**	**0.117**	**0.426**
VAS	0.004	−0.002	0.011	0.004	−0.001	0.009	**0.005**	**0.000**	**0.009**
Married	**−0.467**	**−0.694**	**−0.240**	**−0.377**	**−0.552**	**−0.197**	−0.221	−0.422	0.019
Highest Education	0.066	−0.166	0.298	0.078	−0.092	0.247	0.045	−0.12	0.218
SLE	**−0.028**	**−0.038**	**−0.018**	**−0.028**	**−0.035**	**−0.021**	**−0.028**	**−0.035**	**−0.021**
Living together				0.312	−0.091	0.531	**0.369**	**0.140**	**0.595**
Children							**−0.343**	**−0.541**	**−0.149**

Bold print indicates statistical significance (based on 95% CI)

The three regression analyses performed per TTO question per responder characteristic showed that only two variables from the long list of additional demographic, health, and expectations variables were statistically significantly associated with TTO answers: the dummy variable (yes/no) for living together (but not married) and the dummy variable (yes/no) for having children.

Table 3 shows the results of the model including the variable “living together” (model 2). The results show that age (negative), male gender (positive), married (negative), and SLE (negative) remained statistically significantly associated with years traded off. Only the coefficient for being married changed meaningfully.

Table 3 shows the results of a model adding the variable “having children” to the model (model 3). Age, gender and SLE remained significantly associated with years traded off and their coefficients had the same sign as before. The variable VAS now also showed a significant (positive) association with years traded off, while having children was negatively associated with years traded off. Respondents with children traded off fewer years than those without, therefore. Interestingly, marital status lost significance while people living together significantly traded off more years than others. (A model including a single dummy variable ‘having a partner’ - encompassing both “married” and “living together” - showeded this new variable not to be statistically significantly associated with years while all other results remained similar to those presented in Table 3.)

## Discussion

Not many previous studies have studied the association between TTO responses and a wide variety of background characteristics of respondents. The aim of this study was to do so, with a special emphasis on the consideration of significant others. We investigated the association of these variables with the responses to a TTO exercise solved by a large and representative sample from the Dutch general public, valuing three distinct health states and using a 10 year time frame. The results provided some interesting insights. Expanding the regression model to include a broader range of variables showed having children and living together to be significantly associated with TTO scores. Before addressing our results in more detail, some limitations of our study are highlighted.

A first limitation was that we used an online survey for our study. Given the complex and uncommon task respondents need to fulfil in a TTO exercise, one may expect responses in an interview setting to more accurately describe health state preferences (although every method may have its own advantages and disadvantages). It needs noting that successful TTO studies have been performed online before (e.g. [19]). Second, we did not include a separate valuation module for worse than dead health states, since we felt that the current TTO exercise was already cognitively demanding for members of the general public, as previously argued [11, 16]. This may have influenced our results and it remains interesting to see how the variables investigated here would relate to negative valuations of health states. Third, while our study included a broad array of variables, it needs noting that other potentially influential variables (e.g. more attitudinal questions) also remain understudied. This could be investigated in future research. Fourth, the health state valuations observed in our study were higher than the corresponding national EQ-5D tariffs [2]. This may well relate to our operationalization of the TTO method, which has been shown to be influential before [3]. An important difference between the EuroQol protocol and this study is the number of iterations in the choice method used to obtain the indifference point. Here, we only used one iteration. A more intensive iteration procedure may help respondents to reach their indifference point more accurately and could result in more variation in the answers. It needs noting that the results from this study are closer to the corresponding national EQ-5D tariffs than previous studies which did not use choice based methods [10, 16] More research in the relative advantages of different preference elicitation techniques remains warranted, also in the field of health [20].

Notwithstanding these limitations and areas for further research, this study has provided some interesting results. First, when running the regression model including the more commonly included variables, our results resemble those of earlier research. Notably, SLE and age had a significant influence on years traded off as observed before [10, 16]. This suggests that the influence of these characteristics is relatively stable across studies, supporting the generalizability of these and earlier findings.

Previous research classified marital status as being the third most important influence on TTO scores [6]. However, marital status may be strongly related with both having a partner and with having children. This relationship became clear in our analysis. Only introducing the variable ‘living together’ to the model next to ‘being married’ did not affect the the sign and significance of the variable ‘being married’, while ‘living together’ did not reach significance. However, when also adding the variable ‘having children’ to the model, the results changed. The effect and significance of ‘being married’ was taken over by the variable ‘having children’, rendering the ‘being married’ insignificant. This suggests the variable ‘being married’ may proxy ‘having children’ if the latter is not accounted for. Hence, the influence of marriage per se may be less strong than sometimes suggested.

Moreover, an intriguing result of the final analysis is the positive association between years traded off and ‘living together’. While being married commonly was associated with less years traded off (albeit insignificantly so in our final model), living together was associated with significantly more years traded off. The question *why* living together (but not being married) could lead to more years traded off cannot be answered with this study. However, a recent study by Krol and colleagues [15] showed that respondents, when answering TTO questions, exhibit altruistic preferences. That is, they consider the consequences of their choices on significant others. However, two distinct considerations can be distinguished; one focusing on longevity (living longer for the others despite of poor health, for instance not to be missed), the other focusing on quality of life (giving up more years in order not to be a burden for loved ones). A possible explanation for our results could be that people living together may more often focus on quality of life, while being married (and especially having children) may lead to a focus on longevity. Such motivations behind response patterns are an important area for future research.

Respondents with children more often indicated they had a specific moment in time in mind that they would like to reach, while solving the TTO exercise. This moment in time was often related to children and grandchildren (e.g. seeing them grow up, being at a wedding of children, living long enough for them to be old enough to be independent) among respondents with children. Of those without children, many of the reasons revolved around having a family.

Future quality of life expectations were not significantly associated with TTO responses in this study, although they have been shown to influence TTO responses using a time frame linked to full life expectancy [10]. Quality of life at older ages may therefore be less relevant in a TTO using a 10 year time frame, also because the relevant ages normally are not reached within the 10 year time frame.

Concluding, this study has further explored the question which respondent characteristics influence TTO scores. Next to age, gender and subjective life expectancy, it seems that the variables living together and having children are also influential. The influence of these factors may have been attributed to the variable being married in previous research, but our results suggest the underlying mechanisms to be more diverse and complex. More research into these factors appears warranted in order to improve our understanding of TTO responses.

## Additional files

Additional file 1:
**Health states used.**
Additional file 2:
**Flow of TTO question.**
