# Supplementary material for: “Married with children” the influence of significant others in TTO exercises
Source: Health Qual Life Outcomes. 2015 Jul 2;13:94. doi: 10.1186/s12955-015-0276-7 (PMC4487600; doi:10.1186/s12955-015-0276-7)
Supplement: Additional file 1: — Health states used. [file 12955_2015_276_MOESM1_ESM.docx]

**Appendix 1 Health states used**

11111 (perfect health):

I have **no problems** walking about

I have **no problems** with self-care

I have **no problems** with performing my usual activities

I have **no** pain or discomfort

I am **not** anxious or depressed

21211:

I have **some problems** walking about

I have **no problems** with self-care

I have **some problems** with performing my usual activities

I have **no** pain or discomfort

I am **not** anxious or depressed

22221:

I have **some problems** walking about

I have **some problems** with self-care

I have **some problems** with performing my usual activities

I have **moderate** pain or discomfort

I am **not** anxious or depressed

33312:

I am **confined** to bed

I am **unable** to wash or dress myself

I am **unable** to perform my usual activities

I have **no pain** or discomfort

I am **moderately** anxious or depressed
