# Supplementary material for: “Married with children” the influence of significant others in TTO exercises
Source: Health Qual Life Outcomes. 2015 Jul 2;13:94. doi: 10.1186/s12955-015-0276-7 (PMC4487600; doi:10.1186/s12955-015-0276-7)
Supplement: Additional file 2: — Flow of TTO question. [file 12955_2015_276_MOESM2_ESM.docx]

**Appendix 2: Flow of TTO question**

In the TTO exercise, respondents received three questions for each health state they were asked to value. The health states were provided in the order they were ranked.

[*Introduction*]

Health is valued differently by people. Some people prefer to live shorter but completely healthy, while others prefer to live longer despite poorer health. For three of the five health states that you just rated you will be asked to choose between living another 10 years in that health state or living shorter but in perfect health. We start with the health you rated as the best, and end with the one you rated as the worst.

[*Question 1*]

Imagine you can choose between the following two options:

| *Box with the imperfect health state* |  | *Box with the perfect health state* |
| --- | --- | --- |
| I have **some problems** walking about  I have **no problems** with self-care  I have **some problems** with performing my usual activities  I have **no** pain or discomfort  I am **not** anxious or depressed |  | I have **no problems** walking about  I have **no problems** with self-care  I have **no problems** with performing my usual activities  I have **no** pain or discomfort  I am **not** anxious or depressed |
| **OPTION A**:  You live another 10 years in the health state above, without any change, and then you die. |  | **OPTION B**:  You live another 10 years in the health state above (perfect health), without any change, and then you die. |

Which option do you prefer?

🞏 OPTION A

🞏 BOTH OPTIONS ARE EQUALLY GOOD

🞏 OPTION B

[*If option A was chosen, respondent received the follow-up question below, else skip to question 2.*]

Are 10 years living in poorer health (Option A) better than 10 years living in perfect health (Option B)?

🞏 Yes [*If yes, skip to question 2.*]

🞏 No [*If no, back to question 1.*]

[*Question 2*]

Imagine you can choose between the following two options:

| *Box with the imperfect health state* |  | *Box with the perfect health state* |
| --- | --- | --- |
| I have **some problems** walking about  I have **no problems** with self-care  I have **some problems** with performing my usual activities  I have **no** pain or discomfort  I am **not** anxious or depressed |  | I have **no problems** walking about  I have **no problems** with self-care  I have **no problems** with performing my usual activities  I have **no** pain or discomfort  I am **not** anxious or depressed |
| **OPTION A**:  You live another 10 years in the health state above, without any change, and then you die. |  | **OPTION B**:  You live another 5 years in the health state above (perfect health), without any change, and then you die. |

Which option do you prefer?

🞏 OPTION A

🞏 BOTH OPTIONS ARE EQUALLY GOOD

🞏 OPTION B

[*If both options are equally good was chosen, respondent received the follow-up question below, else skip to question 3.*]

Do you find living 5 years in perfect health (Option B) equally good as living 10 years in poorer health (Option A)?

🞏 Yes [*If yes, skip to question 3.*]

🞏 No [*If no, back to question 2.*]

[*Question 3*]

Suppose you can choose between living 10 years in poorer health or less than ten years in perfect health. In both cases you die afterwards.

| *Box with the imperfect health state* |  | *Box with the perfect health state* |
| --- | --- | --- |
| I have **some problems** walking about  I have **no problems** with self-care  I have **some problems** with performing my usual activities  I have **no** pain or discomfort  I am **not** anxious or depressed |  | I have **no problems** walking about  I have **no problems** with self-care  I have **no problems** with performing my usual activities  I have **no** pain or discomfort  I am **not** anxious or depressed |
| **OPTION A**:  You live another 10 years in the health state above, without any change, and then you die. |  | **OPTION B**:  You live less than X years in the health state above (perfect health), without any change, and then you die. |
|  |  | *Note: X=10 if respondent chose option A in question 2; X=5 if respondent chose option B in question 2.* |

Please indicate on the scale below the minimum number of years that you would like to live in option B in order to make it equally good to option A. How many years in perfect health (option B) would make both options equally good?

[*Respondents were presented with a visual analogue scale ranging from 0 to X, with X=10 if respondent had chosen option A in question 2 and X=5 if respondent had chosen option B. The label of the scale was ‘years’. Below the scale the following text was printed: “I find both options equally good when I’d live another … years in option B” (with the scale value displayed on the dots, at 1 decimal level)*].

[*After the respondent indicated a value, the following question was asked.*]

Do you really find living 10 years in poorer health (Option A) equally good as living Y years in perfect health (Option B)?

🞏 Yes [*If yes, skip to next question .*]

🞏 No [*If no, back to question 3.*]
